# Administration of USP7 inhibitor P22077 inhibited cardiac hypertrophy and remodeling in Ang II-induced hypertensive mice

**DOI:** 10.3389/fphar.2022.1021361

**Published:** 2022-10-25

**Authors:** Yu-Hui Gu, Kai-Wen Ren, Yu Wang, Shi-Hao Wang, Xiao-Hong Yu, Li-Wen Xu, Hui-Hua Li, Hai-Lian Bi

**Affiliations:** ^1^ Institute of Cardiovascular Diseases, First Affiliated Hospital of Dalian Medical University, Dalian, China; ^2^ Department of Cardiology, First Affiliated Hospital of Dalian Medical University, Dalian, China; ^3^ Department of Obstetrics, Dalian Maternal and Child Health Institute, Dalian, China; ^4^ Department of Emergency Medicine, Beijing Key Laboratory of Cardiopulmonary Cerebral Resuscitation, Beijing Chaoyang Hospital, Capital Medical University, Beijing, China

**Keywords:** cardiac remodeling, USP7 inhibitor, P22077, inflammation, oxidase stress

## Abstract

Hypertension is one of the common causes of pathological cardiac hypertrophy and a major risk for morbidity and mortality of cardiovascular diseases worldwide. Ubiquitin-Specific Protease 7 (USP7), the first identified deubiquitinating enzymes, participated in a variety of biological processes, such as cell proliferation, DNA damage response, tumourigenesis, and apoptosis. However, its role and mechanism in cardiac remodeling remain unclear. Here, our data indicated that USP7 expression was increased during Ang II-induced cardiac hypertrophy and remodeling in mice and humans with heart failure, while the administration of its inhibitor p22077 attenuated cardiac hypertrophy, cardiac fibrosis, inflammation, and oxidase stress. Mechanistically, the administration of p22077 inhibited the multiple signaling pathways, including AKT/ERK, TGF-β/SMAD2/Collagen I/Collagen III, NF-κB/NLRP3, and NAPDH oxidases (NOX2 and NOX4). Taken together, these findings demonstrate that USP7 may be a new therapeutic target for hypertrophic remodeling and HF.

## Introduction

Pathological cardiac hypertrophy, characterized by increasing cardiomyocyte size, impaired Ca^2+^ handling, gap junction (GJ) dysfunction, mitochondrial dysfunction, induction of fetal gene program, fibrosis, and cardiac contract dysfunction, is a major risk factor for heart failure (HF) (McMullen and Jennings, 2007; Tham et al., 2015). Compelling evidence demonstrates that Angiotensin II (Ang II) acts as an important stimulatory factor of pathological cardiac hypertrophy and plays an important role in the progression of cardiac hypertrophy and cardiac remodeling. Ang II couples to its receptors, activates Renin-Angiotensin-Aldosterone System (RAAS) and its downstream signaling pathways, such as PI3K/AKT/mTOR, JAK/STAT, and TGF-β/Smad pathways, and subsequently promotes hypertension, cardiac hypertrophy, cardiac fibrosis, and cardiac dysfunction (Mehta and Griendling, 2007). Moreover, Ang II could also activate the inflammation and oxidative stress in cardiac remodeling, which are common pathological alterations in cardiac remodeling, *via* activation of nuclear factor-κB (NF-κB) signaling or NADPH oxidases (NOX2 and NOX4). In addition, overexpression and over-activation of some of these signaling pathway proteins were found in Ang II-induced cardiac hypertrophy and cardiac remodeling, suggesting that maintaining the imbalance between protein synthesis and protein degradation of some harmful proteins was an important therapeutic strategy to protect the heart from cardiac remodeling and heart failure.

The ubiquitin-proteasome system (UPS) plays an important role in maintaining cardiovascular system homeostasis, which selectively ubiquitinylates and triggers the degradation of the misfolded, oxidized, or damaged proteins involved E1s, E2s, E3s, proteasome and deubiquitinating enzymes (DUBs) to ensure the quality and quantity control of proteins in the heart. Ubiquitin (Ub)-specific proteases (UPSs), the largest subfamily of DUBs, remove Ub chains from the substrates to regulate the stability, activity and subcellular localization of the target substrates (Lee et al., 2013; Eletr and Wilkinson, 2014). Increasing evidence has demonstrated that DUBs have a crucial role in cardiovascular diseases, such as cardiac hypertrophy, myocardial infarction, atrial fibrillation, heart failure, and others (Liu et al., 2016; Bi et al., 2020a; Bi et al., 2020b; Hu et al., 2021). USP7 (also called Herpesvirus-associated ubiquitin-specific protease, HAUSP), the first identified DUBs, participated in cell proliferation, DNA damage response, tumourigenesis, inflammation, and apoptosis by regulating its substrates, such as MDM2/p53, PTEN, FOXO4, NF-κB, and other target proteins (Khoronenkova et al., 2012; Smits and Freire, 2016; Xu et al., 2022). Recently, two studies found that the expression of USP7 was significantly increased under hypoxia in cardiomyocytes, while its inhibition or knockdown of USP7 can protect the heart from hypoxia-induced cardiomyocyte injury or myocardial ischemia/reperfusion injury (Xue et al., 2021; Xu et al., 2022). In addition, our previous study using microarrays of gene expression has reported that USP7 is upregulated in Ang II-induced hypertrophic heart in mice, especially on day 7 (Bi et al., 2020a), suggesting that USP7 may involve in cardiac hypertrophy. However, little was known about the role and the molecular mechanism of USP7 in Ang II-induced cardiac remodeling.

Here, our study showed that inhibition of USP7 activity with selective small molecules, p22077, alleviated Ang II-induced hypertrophy, fibrosis, inflammation and oxidase stress, and protected the process from adapted hypertrophy to maladapted remodeling. Mechanically, the administration of USP7 inhibitor p22077 down-regulates multiple signal pathways, which are involved in cardiac hypertrophy and remodeling. In addition, our studies demonstrate that USP7 contributes to the pathogenesis of cardiac hypertrophy and suggest that USP7 could be a new therapeutic target for hypertrophic diseases.

## Materials and methods

### Study subjects

A total of 33 patients diagnosed with HF and 37 normal controls who were admitted to the First Affiliated Hospital of Dalian Medical University from March 2021 to March 2022 were recruited into the present study. The patients with HF were defined according to the recommendation of the ESC with minor modifications (Heidenreich et al., 2022): patients aged at least 18 years with symptomatic heart failure of New York Heart Association (NYHA) functional classes II to IV, BNP ≥ 150 pg/ml) and EF < 50%. The normal controls with normal cardiac function, and no obvious abnormalities in physical examination (routine examination, clinical examination, lab reports, and B-ultrasonography reports), were also recruited. Our study excluded patients with congenital heart disease, primary pulmonary hypertension, pericardial disease, malignancies, recent coronary bypass surgery, and severe valvular heart disease, chronic obstructive pulmonary disease (COPD), hyperthyroidism, hematological system diseases, surgery, or trauma within 2 years, acute and chronic infectious diseases, and significant renal dysfunction (estimated glomerular filtration rate <30 ml/min per 1.73 m^2^). Experimental protocols were approved by the ethics committee of the First Affiliated Hospital of Dalian Medical University, and patients were given informed consent for this study. Participants gave their informed consent and the study was approved by the ethics committee of the First Affiliated Hospital of Dalian Medical University. The blood samples were collected from patients with HF and normal controls for ELISA.

The study contained three male patients (median age = 48, EF = 20 ± 5%, *n* = 3) and three age- and gender-matched controls (median age = 46) as previously described (Xie et al., 2019; Bi et al., 2020a). Samples from patients with HF were obtained from Beijing Anzhen Hospital of the Capital University of Medical Sciences at the time of cardiac transplantation. Normal hearts (non-failing hearts as controls) were obtained from donors with normal cardiac contractile function based on echocardiography of those who had died from motor vehicle accidents. The study was performed in accordance with the Declaration of Helsinki and was approved by the Institutional Ethics Committee of First Affiliated Hospital of Dalian Medical University. Patients provided written consent. Heart tissues were fixed in neutral buffered formalin solution, embedded in paraffin, and prepared for histological examinations.

### Animal and treatment

All the experiments in this study were approved by the Animal Care and Use Committee of Dalian Medical University. The investigation conformed to the Guide for the Care and Use of Laboratory Animals published by the United States National Institutes of Health (NIH Publication No.85-23, revised 1996).

Male wild-type (WT) C57BL/6 mice (8–10 week old), obtained from The Jackson Laboratory (Bar Harbor, ME, United States), were administered intraperitoneally with USP7 inhibitor p22077 (15 mg/kg/day; S7133, Selleck Chem, United States) or DMSO (control) for 1 week, and then were infused with Ang II (1,000 ng/kg/min; A107852; Aladdin, Shanghai, China) or Saline for subsequent 2 weeks using osmotic mini-pumps (Alzet model 1,002; Durect, Cupertino, CA, United States). All mice were anaesthetized with intraperitoneal injection of 2.5% tribromoeothanol (0.02 ml/g; Sigma-Aldrich, St. Louis, MO, United States). The hearts were removed and prepared for further histological and molecular analysis. The heart weight (HW), body weight (BW) and tibial length (TL) were measured, and the HW/BW and HW/TL ratios were calculated.

### Primary cell isolation and culture

Neonatal rat cardiomyocytes (NRCMs) were enzymatically isolated from 1- to 3-day-old Sprague-Dawley rat hearts as described previously (Bi et al., 2020a). Briefly, the ventricle tissues were cut into about 1–3 mm^2^ pieces, digested in trypsin (25200056; Thermo Fisher, Waltham, MA, United States) at 37°C by continually stirring the tissues, and collected into DMEM/F-12 (SH30023.01B; Hyclone, South Logan, UT, United States) containing 25% FBS (16140071; Gibco, Grand Island, NY, United States) every 3–5 min for 8–10 times. The cells were collected and incubated for 90 min at 37°C. Then, the cell suspension was collected and centrifuged at 1,000 rpm for 10 min, and then cultured in DMEM/F12 with 10% FBS, 1% penicillin/streptomycin and 100 mM BrdU. After 36 h incubation, the cells were serum starved for 12 h and then treated with Ang II (100 nM) or Saline for 24 h.

### Blood pressure measurement

The blood pressure of the mice was measured at every 2 days at the same time in the morning after Ang II infusion with a tail-cuff system (BP-2010, Softron, Tokyo, JPN) on a preheated plate at 37°C, as described previously (Shu et al., 2018; Bi et al., 2020b).

### Echocardiographic measurement

Mice were anaesthetized with 1.5% isoflurane (Sigma-Aldrich). Cardiac function was measured by echocardiography in all mice at day 14 after saline or Ang II infusion using a 30 MHz probe (Vevo 770 system; VisualSonics, Toronto, Ontario, Canada) (Xie et al., 2019). Left ventricular (LV) ejection fraction (EF%) and LV fractional shortening (FS%) were calculated based on LV end-diastolic diameter (LVIDd) and LV end-systolic diameter (LVIDs) obtained from M-mode ultrasound (Bi et al., 2020a).

### Histological analysis

The heart tissues were fixed in 4% paraformaldehyde for 1–3 days, embedded in paraffin and then sectioned into 4 µm thick. The sections were stained with a H&E staining kit (D006; Nanjing Jiangcheng Bio Inc., Nanjing, China) and a Masson’s trichrome staining kit (D026; Nanjing Jiangcheng Bio Inc.). Immunohistochemistry was performed with anti-USP7 (1:100; 26948-1-AP; Proteintech Group Inc., Wuhan, Hubei, China), anti-BNP (1:100; 13299-1-AP; Proteintech Group Inc.), anti-α-SMA (1:150; 55135-1-AP; Proteintech Group Inc.), anti-Collagen I (1:100; 14695-1-AP; Proteintech Group Inc.), ant-CD68 (1:150; 28058-1-AP; Proteintech Group Inc.), anti-NLRP3 (1:100; 19771-1-AP; Proteintech Group Inc.), or F4/80 (1:80; 27044-1-AP; Proteintech Group Inc.) antibodies. The heart sections were stained with wheat germ agglutinin (WGA, 50 μg/ml in 1 × PBS, Vector’s Laboratories, Burlingame, CA) for 60 min to evaluate cross-sectional area of myocytes. Frozen sections (5 μm thick) were stained with dihydroethidium (DHE) (BMD00001; Abbkine, Wuhan, Hubei, China) at a dose of 1 mM in PBS at 37°C for 30 min. Digital images were taken at ×200 magnifications of over 10 random fields per slide (ECLIPSE Ni-U,Nikon Instruments Inc, Tokyo, JPN) and analyzed by ImageJ.

### Quantitative real‐time PCR analysis

Total RNA was isolated from fresh heart tissues using TRIzol (Invitrogen/Thermo Fisher Scientific, Carlsbad, CA, United States) according to the manufacturer’s protocol. First-strand cDNA was synthesized from total RNA (1–2 μg) using Random primers/Oligo (dT)-primer mix RT kits (11141ES60; Yeasen, Shanghai, China). The mRNA levels of USP7, atrial natriuretic peptide (ANP), brain natriuretic peptide (BNP), collagen I, collagen III, IL‐1β, IL‐6, NOX2, and NOX4 were determined by real-time qPCR analysis on an Applied Biosystems 7,500 Real-Time PCR System using SYBR Green (RR820A; Takara Bio Inc., Shiga, Japan) as described previously. The values were normalized to those of glyceraldehyde‐3‐phosphate dehydrogenase (GAPDH). Primers were purchased from Sango Biotech (Shanghai, China). Primer sequences were provided in Table 1.

**TABLE 1:** The primers were used in qPCR.

Gene	Forward primer	Reverse primer
USP7	CCA​CAA​GGA​AAA​CGA​CTG​GG	GTA​ACA​CGT​TGC​TCC​CTG​ATT
ANP	CAC​AGA​TCT​GAT​GGA​TTT​CAA​GA	CCT​CAT​CTT​CTA​CCG​GCA​TC
BNP	GAA​GGT​GCT​GTC​CCA​GAT​GA	CCA​GCA​GCT​GCA​TCT​TGA​AT
Collagen I	AGT​CGA​TGG​CTG​CTC​CAA​AA	AGC​ACC​ACC​AAT​GTC​CAG​AG
Collagen III	TCC​TGG​TGG​TCC​TGG​TAC​TG	AGG​AGA​ACC​ACT​GTT​GCC​TG
IL-1β	TGA​AAA​CAC​AGA​AGT​AAC​GTC​CG	CCC​AGG​AGG​AAA​TTG​TAA​TGG​GA
IL-6	TTC​CAT​CCA​GTT​GCC​TTC​TTG	TTG​GGA​GTG​GTA​TCC​TCT​GTG​A
NOX2	CTT​CTT​GGG​TCA​GCA​CTG​GC	GCA​GCA​AGA​TCA​GCA​TGC​AG
NOX4	CTT​GGT​GAA​TGC​CCT​CAA​CT	TTC​TGG​GAT​CCT​CAT​TCT​GG

### Immunoblotting analysis

Protein lysates were extracted from heart tissues using radio immunoprecipitation assay buffer (Solarbio Science Technology Co., Beijing, China), and the concentration was determined using a BCA (P0010; Beyotime, Shanghai, China) protein assay. Equal amounts of protein (40–60 μg) were separated by 8%–12% sodium dodecyl sulfate polyacrylamide gel electrophoresis and transferred to a polyvinylidene difluoride membrane (ISEQ00010; Millipore, Shanghai, China), which was incubated with primary and second antibodies. All blots were developed using a chemiluminescent system, and signal intensities were analyzed using a Gel-Pro 4.5 Analyzer (Media Cybernetics, Rockville, MD, United States), and normalized to Tubulin levels.

### Antibodies

The antibodies used in immunoblotting analysis are as follows: USP7 (1:500; 26948-1-AP), TGF‐β1 (1:1,000; 21898-1-AP), NLRP3 (1:1;000; 19771-1-AP), Tubulin (1:3,000; 66031-1-Ig), NOX2 (1:1,000; 19013-1-AP), NOX4 (1:1,000; 14347-1-AP), collagen I (1:1,000; 14695-1-AP), and collagen III (1:1,000; 22734-1-AP) were purchased from Proteintech Group Inc., (Wuhan, Hubei, China). p-AKT (1:1,000; #9271), AKT (1:1,000; #9272). ERK (1:1,000; #9102), Smad2 (1:1,000; #5339), p65 (1:1,000; #4764) were acquired from Cell Signaling Technologies (Danvers, Massachusetts, United States). p‐p65 (1:500; 310013) was got from Zen Bioscience (Chengdu, Sichuan, China). p‐Smad2 (1:1,000; ab280888) was bought from Abcam (Cambridge, MA, United States). p-ERK (1:500; WLP1512) was obtained from Wanleibio (Shenyang, Liaoning, China). Anti-mouse (1:5,000; A0216) or anti‐rabbit (1:3,000; A0208) IgG secondary antibodies were purchased from Beyotime (Shanghai, China).

### Enzyme-linked immunosorbent assay

Serum levels of total USP7 were determined using human USP7 ELISA kits (FS11681; Westang Bio Inc., Shanghai, China) according to manufacturer instructions.

### Statistical analysis

Statistical calculations were analyzed using Graphpad Prism 8.0 and the SPSS 24.0 software package. A normality test was conducted first. If all the groups satisfied the normality criteria and variances between the groups were equal, we applied Student’s *t*-test or one-way ANOVA (with the Bonferroni *post hoc* test) where appropriate; if the above conditions were not met, we used the nonparametric Mann-Whitney *U* test. Statistical differences were determined by *p* < 0.05. Results are presented as the mean ± SD.

Univariable logistic regression and multivariable logistic regression were used to investigate possible factors associated with HF. Three models were analyzed: Model 1, the crude model without covariate adjustment; Model 2, the multivariable model that adjusted for sex and age, and Model 3, the full risk-adjustment model that adjusted potential confounders that were significant on univariate analysis and those known to be associated with HF including age, sex, eGFR, and HDL. In consideration of the time lag whereby gene expression changes occur earlier than the cardiac structural functional change and the potential collinearity between the echocardiographic parameters, these echocardiographic parameters were not included in the multivariate logistic regression model.

## Results

### The expression of ubiquitin-specific protease 7 is upregulated in patients with heart failure

**FIGURE 1:**
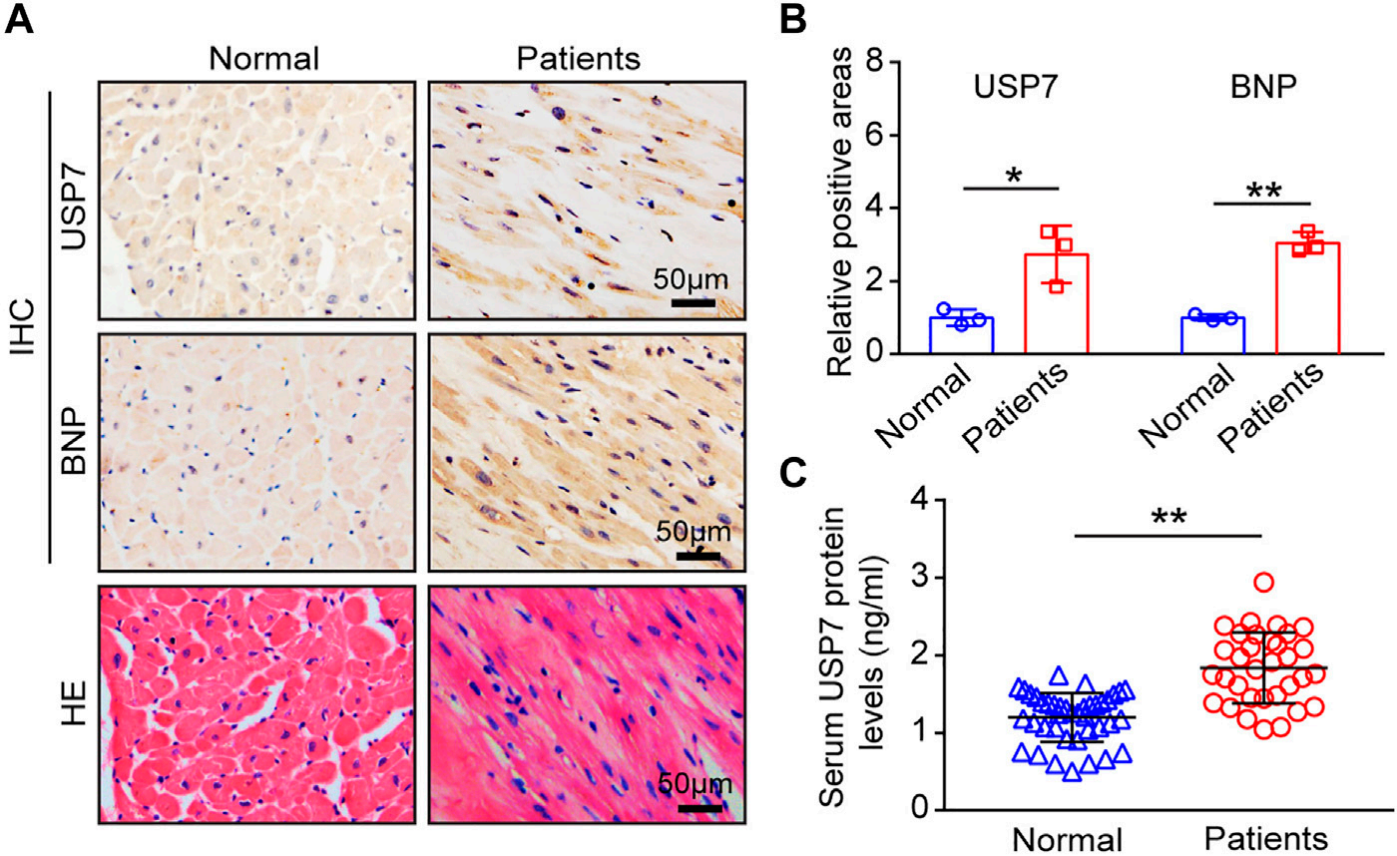
USP7 was overexpressed in patients with heart failure. **(A,B)** Immunohistochemical (IHC) staining of USP7 (upper) and BNP (lower) proteins in the heart tissues from normal control and HF patients (left). Scale bars, 50 μm. Quantification of the relative USP7- and BNP-positive areas (right; *n* = 3). **(C)** The concentration of USP7 protein level in serum from normal controls (*n* = 37) and patients with HF (*n* = 33) was detected by ELISA assay. The data are presented as the mean ± SD, and *n* represents the number of animals per group. *p < 0.05, and **p < 0.01.

To determine the role of USP7 in human with HF, we detected the expression of USP7 both in HF patients (*n* = 3) and normal individuals (*n* = 3) by immunohistochemistry. The expression of USP7 was significantly increased in the hearts from HF patients compared with normal controls (Figures 1A,B). Meanwhile, the expression of brain natriuretic peptide (BNP, a marker of HF) in the hearts from patients with HF was also significantly higher than those in normal controls (Figures 1A,B). Further, the serum concentration of USP7 was tested by a human USP7 Elisa kit, while the USP7 concentration was higher in HF patients compared with that in normal controls (Figure 1C; Table 2, 37 with normal controls and 33 with HF). Moreover, the basic clinical data of normal controls and patients with HF was shown in Table 2. We then evaluated the association of HF and serum USP7 by performing univariable and multivariable logistic regression analyses (Table 3). After being adjusted for the aforementioned confounding factors in model 3, we found that the elevated level of USP7 in serum was an independent predictor of HF. Specifically, the odds ratio (OR) of HF per 1 standard deviation (SD) increase in USP7 was estimated to be 6.250 [95% CI, 1.020–38.313; *p* = 0.048]. These results demonstrated that the abnormal expression of USP7 may be associated with the development of congestive HF.

**TABLE 2:** Clinical characteristics.

Parameters	Normal (*n* = 37)	HF (*n* = 33)	*p*-value
Demographics			
Male sex, *n* (%)	17 (45.94)	19 (57.57)	0.607
Age, years	61.16 ± 14.92	71.06 ± 11.62	0.003**
Hemodynamic variables			
HR, beats/min	75.65 ± 10.38	81.64 ± 17.07	0.077
SBP, mmHg	121.27 ± 10.82	125.33 ± 20.39	0.294
DBP, mmHg	77.97 ± 9.27	79.45 ± 14.64	0.611
Echocardiography			
LVEF, %	59.51 ± 1.87	36.76 ± 10.49	<0.001**
IVS, mm	9.22 ± 1.03	10.24 ± 1.32	<0.001**
LVEDD, mm	45.14 ± 2.92	57.61 ± 9.66	<0.001**
LAD, mm	33.08 ± 2.81	44.91 ± 5.94	<0.001**
Blood test			
USP7, ng/ml	1.25 ± 0.27	1.51 ± 0.43	0.003*
WBC, 10^9/L	5.91 ± 1.12	6.66 ± 1.72	0.375
eGFR, ml/(min × 1.73 m^2^)	91.27 ± 19.43	68.24 ± 26.23	<0.001**
Total cholesterol, mmol/L	4.66 ± 0.80	4.78 ± 1.41	0.658
LDL, mmol/L	2.67 ± 0.74	2.68 ± 0.90	0.515
HDL, mmol/L	1.25 ± 0.27	1.03 ± 0.35	0.005**
Triglycerides, mmol/L	1.48 ± 0.99	1.37 ± 1.04	0.755
BNP, pg/ml	33.25 ± 26.27	2,308.06 ± 3,873.55	<0.001**
History			
Hypertensive, *n* (%)	NA	23 (65.71)	
Diabetes, *n* (%)	NA	13 (37.14)	
coronary artery disease, *n* (%)	NA	18 (51.43)	
Myocardiopathy, *n* (%)	NA	6 (17.14)	
NYHA class, *n* (%)			
II	NA	8 (22.86)	
III	NA	15 (42.86)	
IV	NA	12 (34.28)	

*p < 0.05. **p < 0.01 vs. normal control; HF, heart failure; HR, heart rate; SBP, systolic blood pressure; DBP, diastolic blood pressure; LVEF, left ventricular ejection fraction; IVS, interventricular septum; LVEDD, left ventricular end diastolic diameter; LAD, left atrial diameter; USP7, Ubiquitin-Specific Protease 7; FBG, fasting blood glucose; WBC, white blood cell; SCr, serum creatinine; LDL, low-density lipoprotein; HDL, high-density lipoprotein; BNP, B-type natriuretic peptide; NA, not applicable; NYHA, New York Heart Association. The parameters are mean (SD) or *n* (%).

**TABLE 3:** Logistic regression analysis of USP7 associated with HF.

USP7, per SD increment	OR (95%CI)	*p*-value
Model 1	8.720 (1.873–40.604)	0.006
Model 2	9.018 (1.692–48.072)	0.010
Model 3	6.250 (1.020–38.313)	0.048

Model 1 was unadjusted, Model 2 was adjusted for sex and age, and Model 3 was additionally adjusted for all variables with *p*-value < 0.05 in the logistic regression analysis including age, sex, eGFR, and HDL.

### The expression of ubiquitin-specific protease 7 is upregulated in angiotensin II-induced cardiac remodeling

**FIGURE 2:**
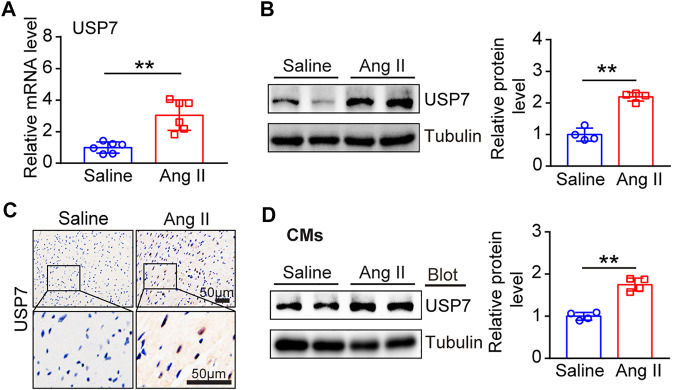
The expression of USP7 was upregulated in Ang II-induced hypertrophic hearts and myocytes. **(A)** qPCR analysis of USP7 mRNA expression in Ang II-infused mouse hearts (*n* = 6). **(B)** Representative immunoblotting analysis of USP7 protein levels in Ang II (1,000 ng/kg/min) -induced hypertrophic hearts (left). Quantification of the relative USP7 protein level (right; *n* = 4). **(C)** Immunohistochemical staining of USP7 in Ang II (1,000 ng/kg/min)-infused mouse hearts (*n* = 6). Scale bar: 50 μm. **(D)** Representative immunoblotting analysis of USP7 protein level in NRCMs exposed to Ang II (100 nM) (left). Quantification of the relative USP7 protein level (right; *n* = 3). The data are presented as the mean ± SD, and n represents the number of animals per group. **p < 0.01.

Next, we determined the changes of USP7 in Ang II-induced hypertrophic heart tissues. Wild-type (WT) mice were treated with Ang II (1,000 ng/kg/min) or saline infusion for 14 days, and then the expression of USP7 was tested. qPCR analysis showed that the expression of USP7 was sharply increased in Ang II-induced hypertrophic heart tissues (Figure 2A). The increased expression of USP7 was also detected at the protein level by immunoblotting analysis (Figure 2B). Furthermore, immunohistochemical staining further demonstrated the increased expression of USP7 in Ang II-infused heart tissues (Figure 2C). Similarly, the protein level of USP7 was significantly augmented in Ang II-treated neonatal rat cardiomyocytes (NRCMs) (Figure 2D). Thus, the increased expression of USP7 indicated that USP7 may participate in Ang II-induced cardiac remodeling.

### Administration of p22077 attenuates angiotensin II-Induced cardiac dysfunction

**FIGURE 3:**
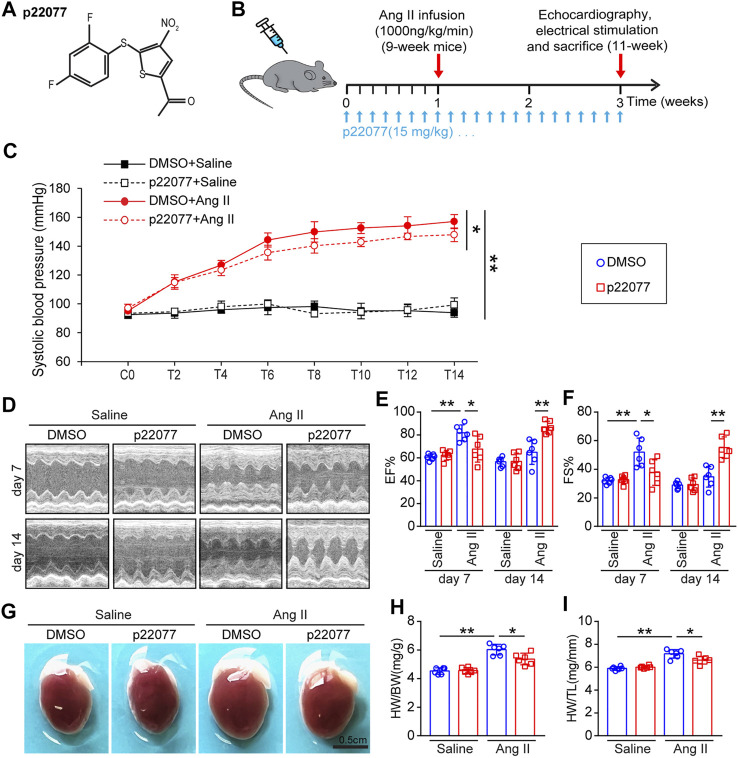
Administration of USP7 inhibitor p22077 attenuates Ang II-induced maladaptive cardiac hypertrophy. **(A)** The basic structure of p22077. **(B)** Protocol for administration of p22077 in a mouse model of cardiac hypertrophy and remodeling. WT mice were treated with p22077 for 7 days and then infused with Ang II or Saline for additional 14 days. **(C)** Tail-cuff method measurement of systolic blood pressure (SBP) every 3 days after Ang II infusion (*n* = 6). **(D–F)** Representative M-mode echocardiography of LV chamber at day 7 and day 14. Measurement of EF% and FS% (*n* = 6 mice per group). **(G–I)** Representative heart sizes from each group. Scale bars, 0.5 cm. The ratios of HW/BW and HW/TL (*n* = 6 per group). *p < 0.05, and **p < 0.01.

To determine the role of USP7 in regulating cardiac remodeling, we used P22077, a potent and selective USP7 inhibitor, which has been identified by activity-based chemical proteomics (Altun et al., 2011; Fan et al., 2013). WT mice were treated with p22077 and Ang II infusion (1,000 ng/kg/min) for 14 days (Figures 3A,B). Administration of p22077 significantly declined Ang II-induced elevation of blood pressure compared with DMSO-treated mice (Figure 3C). Echocardiography indicated that administration of p22077 sharply attenuated Ang II-induced cardiac hypertrophy and cardiac contractile dysfunction, as reflected by delaying the decrease of left ventricular (LV) ejection fraction (EF%) and fractional shortening (FS%), compared with DMSO plus Ang II-treated mice (Figures 3D–F). Moreover, Ang II-induced cardiac hypertrophy was also indicated by an increase in the heart size, HW to BW ratio, and HW to TL ratio (Figures 3G–I). In addition, the expression of USP7 has not been affected by P22077 (Supplementary Figures S1A,B).

### Administration of p22077 attenuates angiotensin II-Induced cardiac hypertrophy and fibrosis

**FIGURE 4:**
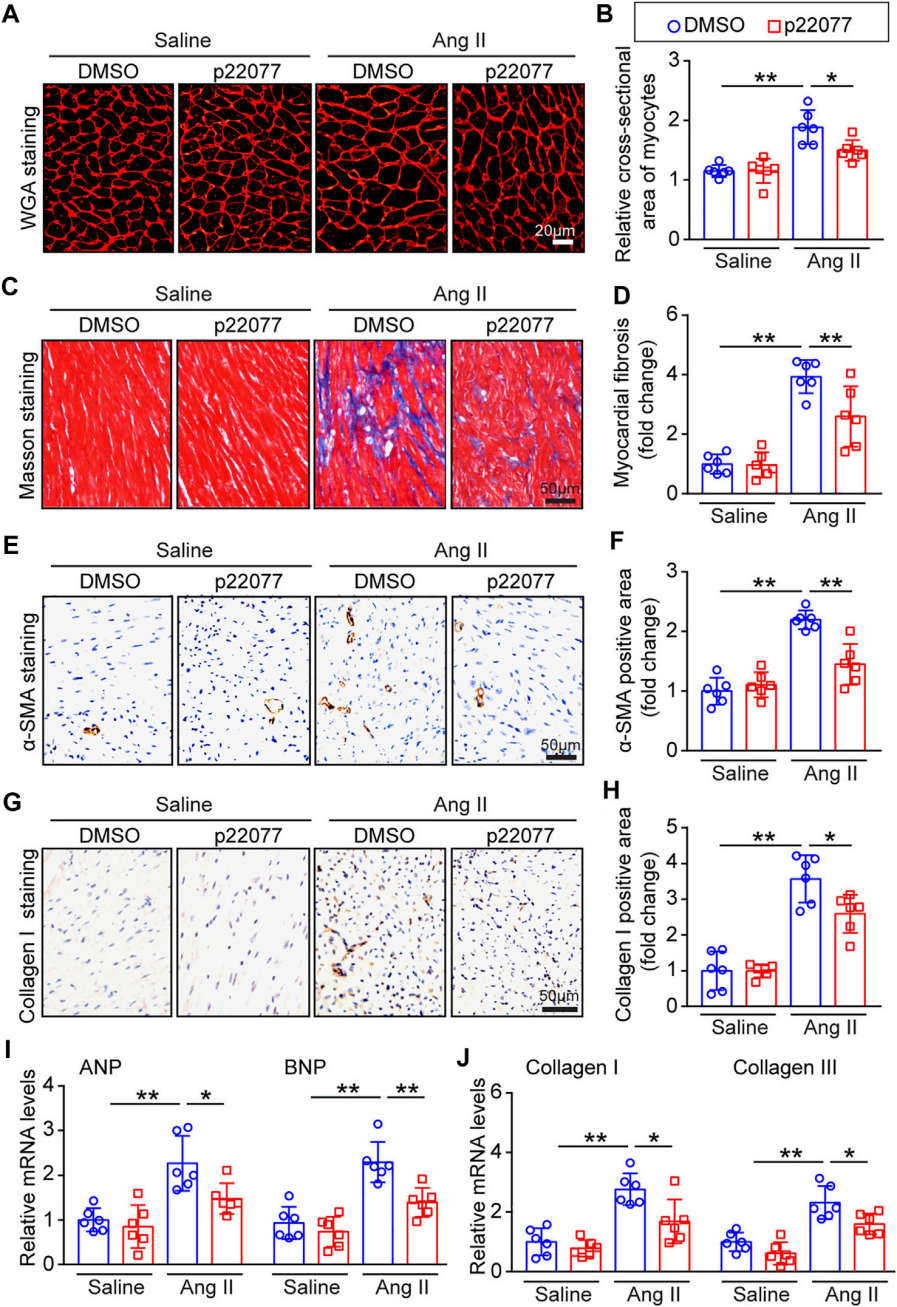
Administration of p22077 attenuates Ang II-induced cardiac hypertrophy and cardiac fibrosis. **(A,B)** Histological examination of cardiac hypertrophy by TRITC-WGA staining (Left) and quantification of the relative myocyte cross-sectional area (200 cells counted per heart, Right; *n* = 6). Scale bars, 20 μm. **(C,D)** Histological examination of myocardial fibrosis detected by Masson’s trichrome staining (Left) and quantification of the relative fibrosis area (Right; *n* = 6). Scale bars, 50 μm. **(E,F)** Representative IHC and quantification of α-SMA-positive area (*n* = 6 mice per group). Scale bars, 50 μm. **(G,H)** Representative IHC and quantification of Collagen I-positive area (*n* = 6 mice per group). Scale bars, 50 μm. **(I,J)** qPCR analysis of ANP, BNP, collagen I and collagen III mRNA levels in the heart tissues. The data are normalized to the GAPDH expression (*n* = 6 per group). The data are presented as the mean ± SD, and *n* represents the number of animals per group. *p < 0.05, and **p < 0.01.

To determine the role of p22077 in regulating cardiac remodeling, we performed WGA staining and Masson staining. As expected, the cross-sectional area of myocytes and the area of fibrosis were significantly increased in Ang II-infusion mice, while these were markedly attenuated in p22077-treated animals (Figures 4A–D). Moreover, the Ang II-induced increase of the α-smooth muscle actin (α-SMA)-positive and collagen I-positive myofibroblasts was decreased in p22077-treated animals compared with control mice (Figures 4E–H). The expression of hypertrophic markers ANP and BNP and the expression of fibrotic markers collagen I and collagen III were markedly attenuated in p22077-treated animals (Figures 4I,J). Similarly, there was no significant difference in these parameters between the two groups after saline infusion (Figures 4A–J). These results suggest that USP7 contributes to Ang II-induced cardiac dysfunction and hypertrophic remodeling.

### Administration of p22077 attenuates angiotensin II-Induced inflammation and oxidative stress

**FIGURE 5:**
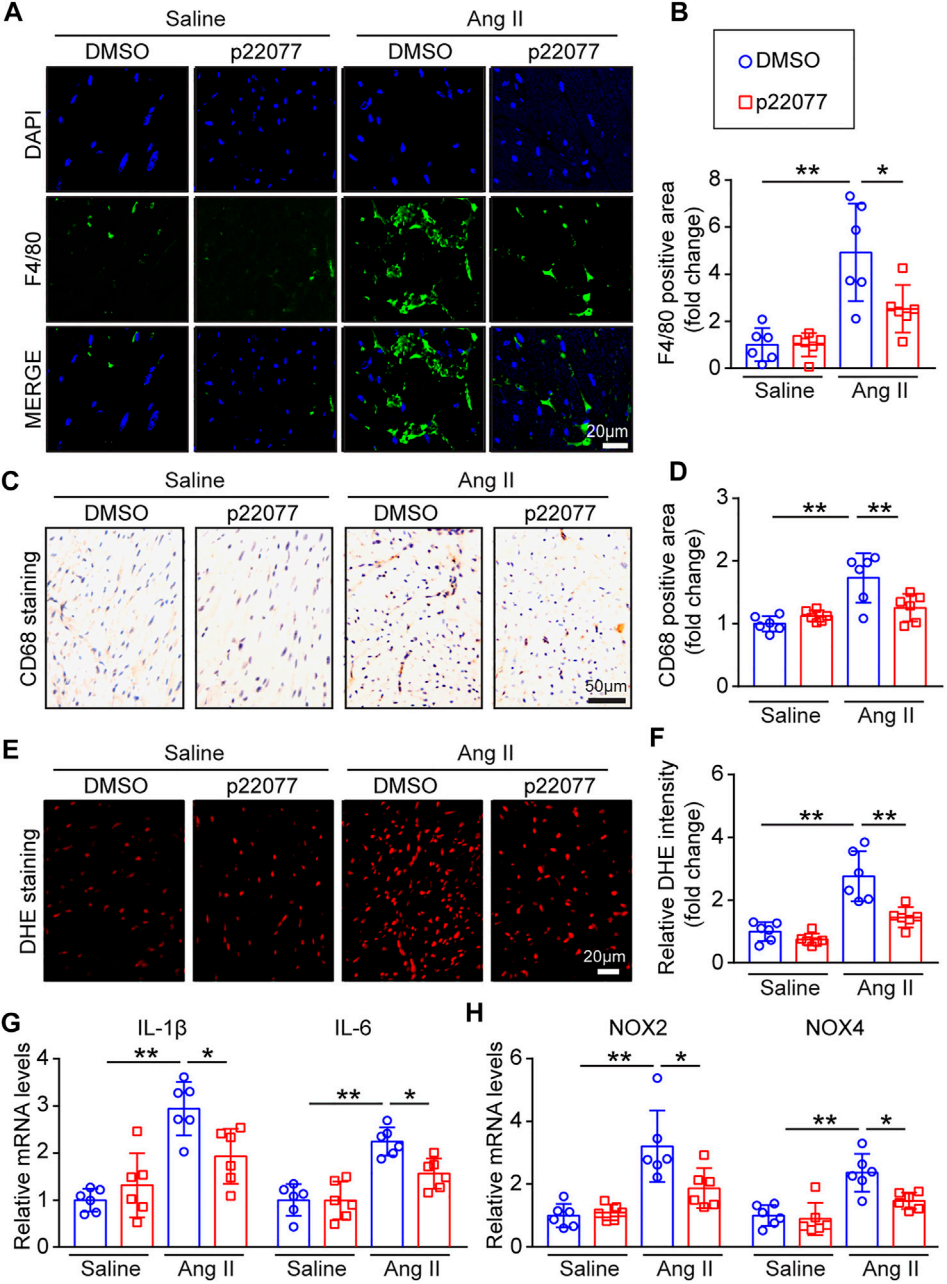
Administration of p22077 attenuates Ang II-induced inflammation and oxidase stress. **(A,B)** Immunofluorescent staining of heart sections for examination of F4/80-positive macrophages (green) and nuclei (DAPI, blue) (*n* = 6). Scale bar = 20 μm. Quantification of F4/80 positive area. **(C,D)** Representative IHC and quantification of CD68-positive area (*n* = 6 mice per group). Scale bars, 50 μm. **(E,F)** Dihydroethidium (DHE) staining of heart sections (left). Quantification of DHE intensity (*n* = 6). Scale bar = 20 μm. **(G,H)** qPCR analysis of IL-1β, IL-6, NOX2, and NOX4 in heart tissues (*n* = 6). The data are presented as the mean ± SD, and n represents the number of animals per group. *p < 0.05, and **p < 0.01.

It has been reported that inflammation and oxidative stress play a critical role in regulating Ang II-induced cardiac remodeling (Crowley, 2014; de Almeida et al., 2020). We further examined the effect of p22077 on the inflammatory response and the production of reactive oxygen species (ROS) in Ang II-induced cardiac hypertrophy. Immunofluorescent assay, IHC assay and DHE staining revealed that Ang II infusion caused a marked increase in the inflammatory cells, NLRP3-inflammasome pathway and superoxide production, including that of F4/80-positive and CD68-positive macrophages, NLRP3-expression and ROS production, in the vehicle-treated mice, but this increase was attenuated in the p22077-treated mice (Figures 5A–F; Supplementary Figures S2A,B). Moreover, the Ang II-induced increase of the mRNA levels of proinflammatory (IL-1β and IL-6, downstream targets of NLRP3-inflammasome activation) and NADPH oxidases (NOX2 and NOX4) were attenuated in p22077-treated mice (Figures 5G,H). These parameters in saline groups treated with and without p22077 have no significant difference (Figures 5A–H). These results suggest that USP7 contributes to Ang II-induced inflammation, oxidative stress and the progression of cardiac remodeling.

### Administration of p22077 reduces multiple signaling pathways

**FIGURE 6:**
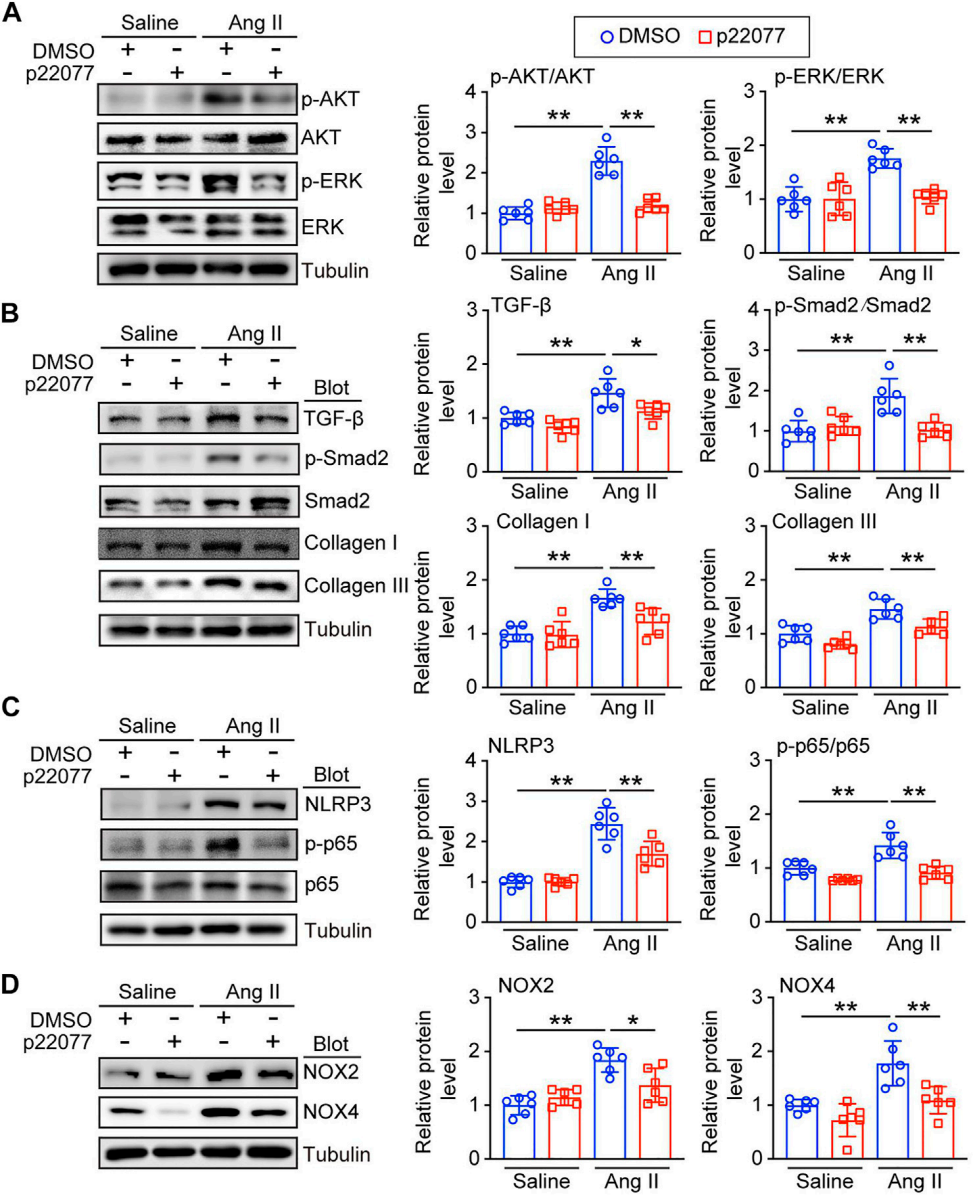
Administration of p22077 regulates multiple signaling pathways in Ang II-induced cardiac remodeling. **(A)** Representative immunoblotting analysis of p-AKT, AKT, p-ERK, ERK, and Tubulin in heart tissues from each group (left). Quantification of the relative protein levels (right, *n* = 6). **(B)** Representative immunoblotting analysis of TGF-β1, p-Smad2, Smad2, Collagen I, Collagen III, and Tubulin (left). Quantification of the relative protein levels by densitometry (right, *n* = 6). **(C)** Representative immunoblotting analysis of NLRP3, p65, p-p65, and Tubulin (left). Quantification of the relative protein levels (right, *n* = 6). **(D)** Representative immunoblotting analysis of NOX2, NOX4, and Tubulin (left). Quantification of the relative protein levels (right, *n* = 6). The data are presented as the mean ± SD, and n represents the number of animals per group. *p < 0.05, and **p < 0.01.

Ang II triggers multiple signaling pathways, such as AKT/mTOR/p-ERK, TGF-β/Smad2/3, NF-κB/NLRP3, and NADPH oxidases, which are involved in hypertrophy, fibrosis formation, inflammation response and oxidase stress (Altun et al., 2011; Crowley, 2014). To determine the molecular mechanism of the protective role of p22077 on Ang II-induced cardiac remodeling, we performed the immunoblotting assay to investigate several signaling pathways associated with cardiac remodeling. Compared with Ang II plus DMSO group, the administration of p22077 attenuated Ang II-induced increased expression or the activation of p-AKT, p-ERK, TGF-β1, p-Smad2, collagen I, collagen III, p-p65, NLRP3, NOX2, and NOX4 in the Ang II plus p22077 group (Figures 6A–D). There was no significant difference in these parameters between the two groups treated with saline (Figures 6A–D).

## Discussion

**FIGURE 7:**
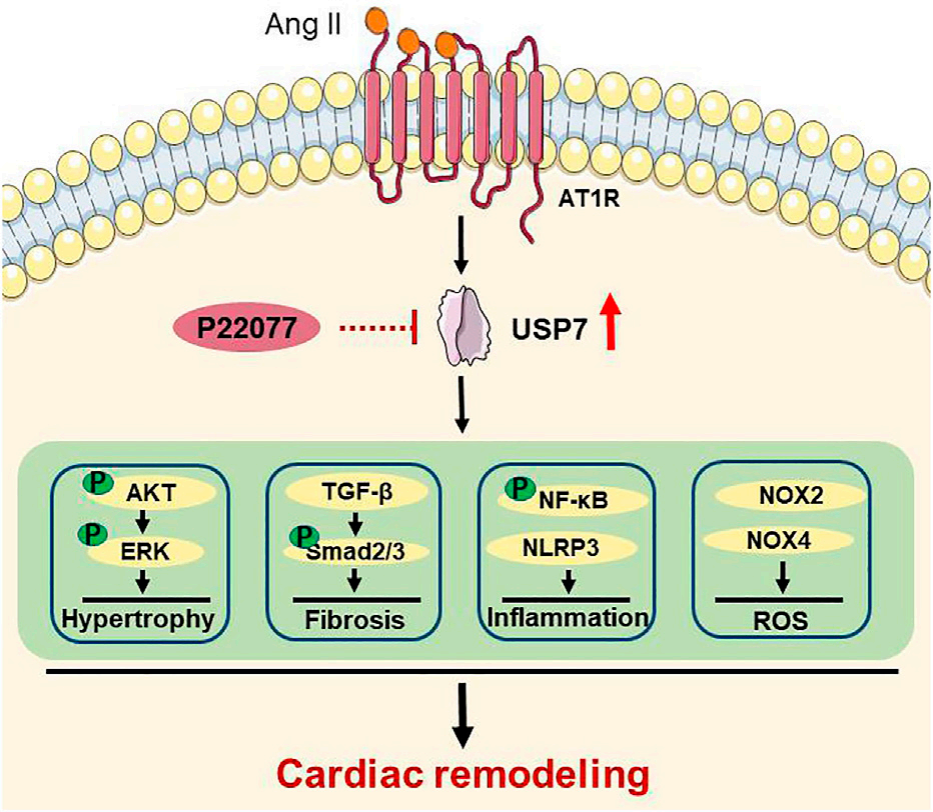
A working model for the administration of USP7 inhibitor p22077 to suppress cardiac remodeling in Ang II-induced hypertensive mice. Hypertension induces the expression of USP7. Blocking USP7 activity by p22077 inhibits multiple signaling pathways, which involved in hypertrophy, fibrosis, inflammation, and oxidative stress, thereby attenuates cardiac remodeling in Ang II-induced hypertensive mice.

In this study, we for the first time demonstrated that the deubiquitinase USP7 has a crucial role in regulating Ang II-induced cardiac hypertrophy and cardiac remodeling. The major findings are as follows: 1) The expression of USP7 was upregulated both in heart tissues and serum from patients with heart failure, and in Ang II-induced hypertrophic mice hearts. 2) The inhibition of USP7 by p22077 significantly reduced the Ang II-induced cardiac hypertrophy, fibrosis, inflammation and oxidative stress, and recovery of cardiac contractile dysfunction. 3) Mechanically, the administration of USP7 inhibitor p22077 reduced the multiple cell signals, including AKT/ERK, TGF-β/SMAD, NF-κB, NOX2, and NOX4, which are associated the cardiac remodeling. Thus, our data indicated that USP7 plays an important role in Ang II-induced cardiac hypertrophy and remodeling and may be a novel target for the treatment of hypertrophic cardiovascular diseases. The proposed working model was shown in Figure 7.

Pathological hypertrophy and remodeling can be stimulated by prolonged and abnormal stress, such as hypertension, pressure overload, and myocardial infarction, which promote the progression from adaptive compensation to maladaptive decompensation and finally heart failure (Opie et al., 2006). Ang II, a powerful effector peptide of the RAAS and a hypertrophic agent, can induce cardiac hypertrophy and maladaptive cardiac remodeling by activating different signaling pathways, such as AKT/ERK, TGF-β/SMAD/collagen I/collagen III, NF-κB/NLRP3, NADPH kinases (NOX2 and NOX4) (Sag et al., 2014; Wang et al., 2019). Increasing evidence demonstrated that oxidase stress plays an important role in cardiac remodeling (Wen et al., 2012). Currently, the increased expression and activity of NADPH oxidases play a central position in ROS production leading to cardiac remodeling, which causes the activation of the hypertrophic signal pathway ERK1/2, AKT/mTOR and inflammation response signaling pathway NF-κB/NLRP3 and dysregulation of cardiac fetal contractile genes (Zhao et al., 2015). Here, our studies provided convincing evidence that the administration of p22077 decreased the Ang II-induced oxidase stress, reflected by inhibiting the ROS production and the expression of oxidative stress response proteins (NOX2 and NOX4) (Figures 5C,D,F). Moreover, the Ang II-induced cardiac hypertrophy, fibrosis and inflammation, which can be activated by ROS, were also attenuated with the treatment of USP7 inhibitor p22077 (Figures 4, 5), suggesting that the protective effect of p22077 on the Ang II-induced cardiac hypertrophy, fibrosis and inflammation may be related to oxidase stress. In addition, the administration of p22077 extremely abolished the Ang II-induced augment of the expression of NOX2 and NOX4 as well as the activity of its target signaling pathway, including ERK1/2, AKT/mTOR and NF-κB/NLRP3 (Figure 6A–D). Meanwhile, previous studies report that USP7 could target and deubiquitylate NOX4 (Liu et al., 2020).

USP7, as an important deubiquitinase, has been reported to participate in different biological activities by stabilizing and deubiquitylating several substrate proteins, such as MDM2, SMAD3, PTEN, NF-κB, Keap1, NLRP3 and others (Colleran et al., 2013; Huang et al., 2021; Xu et al., 2022). A study has shown that the USP7 inhibitor p22077 blocks inflammation response and the activation of NLRP3 inflammasome (Palazon-Riquelme et al., 2018), while activation of NLRP3 inflammasome involved in cardiac remodeling, suggesting that USP7 may play an important role in cardiac remodeling. Moreover, inhibition of USP7 activated p53 and decreased TfR1, and then attenuates ferroptosis and protects the heart from myocardial I/R injury (Tang et al., 2021), suggesting that inhibition of USP7 may be a potential therapy for cardiovascular disease. Consistent with these reports, our data demonstrated that administration of USP7 inhibitor p22077 attenuated the Ang II-induced increase of the inflammation response marker (IL-1β and IL-6) and the F4/80-positive macrophages (Figures 5A,E). Taken together, these results indicate that the USP7 inhibitor p22077 may modulate cardiac remodeling by targeting and stabilizing a series of substrate proteins, which play important roles cardiac hypertrophy, fibrosis, inflammation, oxidase stress, and the progression of heart failure (Figure 7).

There are several limitations in our study. 1) Our previous studies and present study have demonstrated that Ang II upregulated USP7 both in mRNA and protein levels in hypertrophic hearts (Figure 1) (Bi et al., 2020a). However, the upstream mechanism in Ang II-induced increasing expression of USP7 needs to be tested in the future. 2) NOX2 and NOX4 are the two NADPH oxidases in the heart, which are the major generators of ROS. Also, our studies demonstrated that p22077 could significantly down-regulate the protein level of NOX2 and NOX4 (Figure 6). Since both enzymes share sequence homology, and previous study demonstrated that USP7 could target and deubiquitylate NOX4 (Liu et al., 2020), whether USP7 could also target and deubiquitylate NOX2 and whether USP7 regulate the location and the activation of NOX2 in the myocyte need to be studied in further.

## Conclusion

The current study demonstrated that the deubiquitinase USP7 was upregulated in cardiac hypertrophy and the administration of its inhibitor p22077 attenuated Ang II-induced cardiac hypertrophy, fibrosis, inflammation, and oxidase stress, suggesting that USP7 might be a novel therapeutic target and a hypertrophy marker for cardiac hypertrophy and heart failure.

## Data Availability

The original contributions presented in the study are included in the article/Supplementary Material, further inquiries can be directed to the corresponding authors.

## Supplementary material

SUPPLEMENTARY FIGURE S1 The effect of p22077 on the expression of USP7 in Ang II-induced cardiac remodeling. **(A)** Representative immunoblotting analysis of USP7 and Tubulin in heart tissues from each group (left, *n* = 6). **(B)** Quantification of the relative protein levels (right, *n* = 6). The data are presented as the mean ± SD, and *n* represents the number of animals per group. **p < 0.01 and NS, not significant.

SUPPLEMENTARY FIGURE S2 Administration of p22077 attenuates Ang II-induced inflammation. **(A,B)**Representative IHC and quantification of NLRP3-positive area (*n* = 6 mice per group). Scale bars, 50 μm. The data are presented as the mean ± SD, and *n* represents the number of animals per group. ***p* < 0.01.
