# Supplementary figures and images for: Administration of USP7 inhibitor P22077 inhibited cardiac hypertrophy and remodeling in Ang II-induced hypertensive mice
Source: Front Pharmacol. 2022 Oct 25;13:1021361. doi: 10.3389/fphar.2022.1021361 (PMC9640964; doi:10.3389/fphar.2022.1021361)

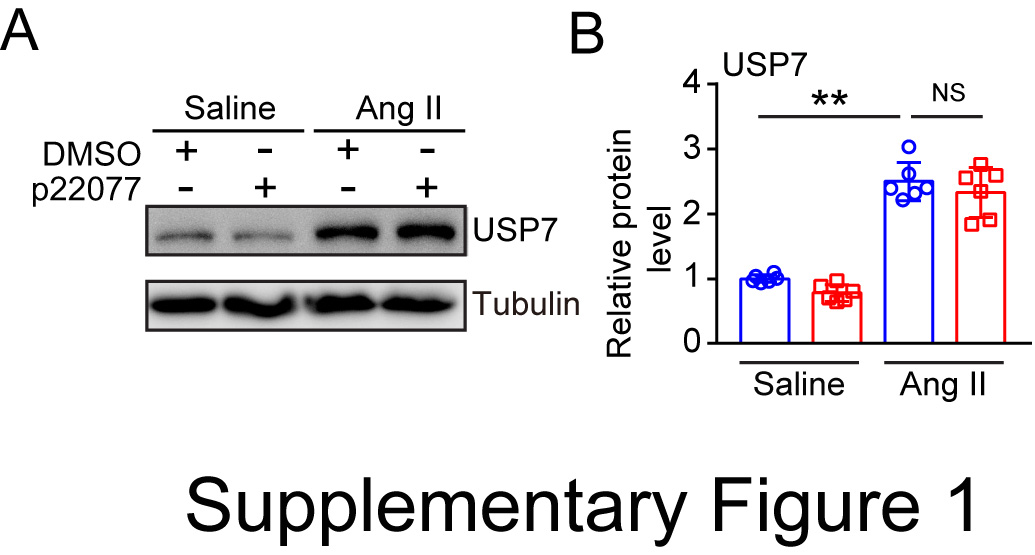

Supplement: Supplementary file 1 [file Image1.jpg]
