# Supplementary material for: Administration of USP7 inhibitor P22077 inhibited cardiac hypertrophy and remodeling in Ang II-induced hypertensive mice
Source: Front Pharmacol. 2022 Oct 25;13:1021361. doi: 10.3389/fphar.2022.1021361 (PMC9640964; doi:10.3389/fphar.2022.1021361)
Supplement: Supplementary file 2 [file DataSheet1.docx]

**Supplementary Figures and Figure Legends**

**Supplementary Figure 1. The effect of p22077 on the expression of USP7 in Ang II-induced cardiac remodeling. (A)** Representative immunoblotting analysis of USP7 and Tubulin in heart tissues from each group (left, n = 6). **(B)** Quantification of the relative protein levels (right, n = 6). The data are presented as the mean ± SD, and n represents the number of animals per group. ***p* < 0.01 and NS: not significant.

**Supplementary Figure 2. Administration of p22077 attenuates Ang II-induced inflammation.** (**A-B**) Representative IHC and quantification of NLRP3-positive area (n = 6 mice per group). Scale bars, 50 μm. The data are presented as the mean ± SD, and n represents the number of animals per group. ***p* < 0.01.
